# Supplementary material for: satuRn: Scalable analysis of differential transcript usage for bulk and single-cell RNA-sequencing applications
Source: F1000Res. 2022 Aug 8;10:374. Originally published 2021 May 11. [Version 2] doi: 10.12688/f1000research.51749.2 (PMC9892655; doi:10.12688/f1000research.51749.2)
Supplement: Peer review history

# Peer review: Reviewer response for version 2

**DOI:** 10.5256/f1000research.136695.r146806

**Published:** 2023-02-01

**Authors:** Marek Cmero

**Affiliations:** 1 Walter and Eliza Hall Institute of Medical Research, Melbourne, Vic, 3052, Australia; 2 Department of Medical Biology, The University of Melbourne, Melbourne, Vic, 3052, Australia

In version 2 of the manuscript, the authors have comprehensively addressed the suggested revisions. There are some remaining minor issues; addressing these would help with clarity:

1. The axis labels for Figure S20A should read "number of samples per group" rather than cells.
2. Table S1 should use decimal points instead of commas for percentage values.
3. There are some mixed-up figure references in the last paragraph in the "Performance on simulated bulk RNA-seq datasets" section (page 11). I believe the first sentence should refer to S9 rather than S13. S10-12 are also not referenced in the text.
4. My original point about Figure 8 referred to the A-C labels that were missing. These labels still haven't been added (A-D labels are also missing for Figure 6).

Is the rationale for developing the new method (or application) clearly explained?

Yes

Is the description of the method technically sound?

Yes

Are the conclusions about the method and its performance adequately supported by the findings presented in the article?

Yes

If any results are presented, are all the source data underlying the results available to ensure full reproducibility?

Yes

Are sufficient details provided to allow replication of the method development and its use by others?

Partly

Reviewer Expertise:

Bioinformatics, method development, transcriptomics

I confirm that I have read this submission and believe that I have an appropriate level of expertise to confirm that it is of an acceptable scientific standard.

# Peer review: Reviewer response for version 2

**DOI:** 10.5256/f1000research.136695.r146805

**Published:** 2023-01-30

**Authors:** Alejandro Reyes

**Affiliations:** 1 Novartis Institutes for BioMedical Research (NIBR), Basel, Switzerland

Thanks to the authors for addressing fully the concerns raised in my first revision. They now discuss the general limitations of scRNA-seq affect differential transcript usage analyses, and how each tool is able to capture events of different magnitudes.  Finally, I want to congratulate the authors for putting the manuscript together and a well-documented and functional software package.

Is the rationale for developing the new method (or application) clearly explained?

Yes

Is the description of the method technically sound?

Yes

Are the conclusions about the method and its performance adequately supported by the findings presented in the article?

Partly

If any results are presented, are all the source data underlying the results available to ensure full reproducibility?

Yes

Are sufficient details provided to allow replication of the method development and its use by others?

Yes

Reviewer Expertise:

Bioinformatics, Computational Biology, Drug Discovery

I confirm that I have read this submission and believe that I have an appropriate level of expertise to confirm that it is of an acceptable scientific standard.

# Peer review: Reviewer response for version 1

**DOI:** 10.5256/f1000research.54945.r85196

**Published:** 2021-06-21

**Authors:** Alejandro Reyes

**Affiliations:** 1 Novartis Institutes for BioMedical Research (NIBR), Basel, Switzerland

The authors propose a new method to test for differences in transcript usage for both bulk RNA-seq and single-cell RNA-seq data. The manuscript is clear and the method is sound and well explained. The authors provide an excellent implementation of their method as a Bioconductor package whose vignette is easy to follow. The main advantage and novelty of satuRn is that it scales well with very large samples, which is a limitation of some of the best performing methods in literature. Below are a couple of suggestions to provide more context to the DTU events on the benchmarks, assess to what extent DTU for single cells might be a limitation and a couple of requests for clarifications in the manuscript.

- The transcript swapping strategy seems quite powerful to benchmark DTU methods. This strategy is probably introducing DTU of different magnitudes. It would be highly informative if the authors can assess the performance of the different methods as a function of the strength of the induced DTU. How is the performance of satuRn and the other tools across different ranges of DTU induction?
- The authors describe a strategy to calculate p-values based on t-test on the estimated log-odds transcript ratios. They also describe that this strategy is too liberal for scRNA-seq analyses and provide an alternative approach that converts these p-values to z-scores and use locfdr to estimate an empirical null distribution. Based on the paper and the vignette, it was unclear to me which strategy is followed for bulk RNA-seq analyses? Does the user have the possibility of choosing which strategy to use?
- The filtering strategy for scRNA-seq datasets is very stringent (at least 1 count in 50% of the cells) and might result in mostly highly expressed genes/transcripts. How many genes can be tested for DTU after such thresholds are applied in real scRNA-seq data? It would also be very informative to show benchmarks with less stringent criteria to assess how the presence of many zeros affect each method. 
- Given that most scRNA-seq protocols only sequence the 3’ end of transcripts, it might be unfeasible to reliably estimate transcript expression (this is discussed in Ntranos *et al. *(2019 ^1^)). Examples would be when the transcripts are not distinguishable from the 3’ends alone. The authors should consider testing their approach and benchmarks at the level of transcript compatibility counts. 
- I found unclear whether stageR applied by default (as I understand from the vignette)? If so, I think it is important to apply stageR to all the methods tested in the benchmarks to distinguish the performance of saturn vs satuRn + stageR. 
- The authors describe the applicability of their method for differential transcript usage. However, as I understand the model, it could also be used for differential exon usage, or groups of reads supporting an alternative splicing event (counts from alternative splicing event A counts from event B, or intron clusters as in leafcutter, etc). I think the authors could showcase these examples to expand the applications of their model.
- In most ROC curves it’s a bit hard to distinguish each different line due to over plotting.

Is the rationale for developing the new method (or application) clearly explained?

Yes

Is the description of the method technically sound?

Yes

Are the conclusions about the method and its performance adequately supported by the findings presented in the article?

Partly

If any results are presented, are all the source data underlying the results available to ensure full reproducibility?

Yes

Are sufficient details provided to allow replication of the method development and its use by others?

Yes

Reviewer Expertise:

Bioinformatics, Computational Biology, Drug Discovery

I confirm that I have read this submission and believe that I have an appropriate level of expertise to confirm that it is of an acceptable scientific standard, however I have significant reservations, as outlined above.

## References

[1] : A discriminative learning approach to differential expression analysis for single-cell RNA-seq. *Nat Methods* .16(2) : 10.1038/s41592-018-0303-9 163-166 10.1038/s41592-018-0303-9 30664774

# Response

**Published:** 2022-07-25

**Authors:** Jeroen Gilis

**Affiliations:** Ghent University - VIB, Belgium

We are grateful for the comments and suggestions of both reviewers, which have markedly improved the readability of the manuscript and have led to several additional analyses and findings. We here first summarise the major and minor changes to the manuscript and then address the reviewer-specific comments point-by-point:

 Major changes:

- We include a case study analysis on single-cell RNA-seq data with a more complex multifactor experimental design. We first show that including additional covariates to the differential transcript usage (DTU) analysis affects the ranking of transcripts in the resulting top list as compared to an analysis without these covariates. Second, we use the more complex model to identify covariate-specific changes in DTU.
- We include a case study that performs a differential usage analysis at the level of equivalence classes. We qualitatively compare results between a transcript-level and equivalence class-level analysis on the same data, highlighting the advantages and disadvantages of both analyses.
- We included a short case study on differential exon usage on a small bulk RNA-seq dataset, comparing satuRn with DEXSeq.
- We elaborate on the effect of data sparsity and filtering on the benchmark performances of the different DTU tools. We additionally summarize the key characteristics of the different datasets.
- We elaborate on the comparison between using raw counts or scaledTPM abundances in the context of DTU analyses.
- We assess the performance of DTU methods as a function of the strength of the induced DTU signal as introduced by the swapping strategy in our performance benchmarks.
- We additionally include a scalability analysis of the different DTU tools on bulk RNA-seq data.

Minor changes:

- We discuss the discrepancy of the performance benchmark results between the dataset from Love *et al.* and the other datasets.
- We add information on the quantification strategies for the single-cell RNA-seq datasets.
- We make more explicit which strategies were used for introducing DTU signal in the different datasets that were used for benchmarking the DTU methods.
- We clarify the acronym FDP.
- We labeled the panels of Figure 8. In addition, we provided labels to Figure 6 and adapted the captions accordingly.
- We provide a more detailed description of the null distribution histograms in the *Extended data* figures.
- We clarify that, by default, satuRn adopts the testing strategy that is based on the empirical null distribution, but that users could opt for working with the unadjusted p-values as well.
- We clarify that we did not perform stage-wise testing adjustment in any of the analyses presented in the manuscript.
- We aimed to improve the layout of the performance curves.

Response to comments Reviewer 2:

1. The swapping strategy will indeed induce DTU signal of different magnitudes. We followed the reviewer’s suggestion of assessing the performance of the different methods as a function of the strength of the induced signal.
  Specifically, we have included four additional figures in the *Extended data* (Figures S16-S19), corresponding to the four benchmarks that used swapping, where we stratify FDR-TPR curves based on the strength of the induced DTU signal. As an estimate for the strength of the signal, we take the difference in the empirical average transcript usage between the two groups of cells/samples. The empirical usage was computed by dividing the transcript-level count with the corresponding gene-level count in each cell/sample. Based on this metric, the FDR-TPR curves are stratified in 6 categories: a difference in transcript usage between groups of 0-10%, 10-20%, 20-30%, 30-40%, 40-50% and 50-100%. A brief discussion of the results is included in the “Performance on real single-cell data” paragraph of the Results section of the manuscript.
  First, as expected, the ability of all methods to detect DTU decreases when the strength of the DTU signal decreases. Second, the difference of transcript usage that can be correctly detected is dependent on the data: small differences in transcript usage can be more easily detected in bulk data as compared to single-cell data, likely owing to the larger sequencing depth and lower variability of bulk datasets. Third, inference improves for all strata when the number of cells or samples increases. Fourth, we observed in all benchmark datasets that satuRn and DoubleExpSeq are more successful in detecting small differences as compared to the other methods. As such, the performance gain of satuRn and DoubleExpSeq in the overall results (without stratification) can be attributed to picking up DTU with smaller effect sizes. As a final note, having different transcript-level filtering procedures (edgeR filter versus DRIMSeq filter) did not affect these findings. Adopting scaledTPM abundances instead of raw counts did not affect the results, either (results not shown).
2. In the second comment, the reviewer asked if satuRn by default uses the empirical null strategy to improve FDR control, if this is also the case in bulk analyses and if this is tunable by the user.
  By default, we indeed always use the strategy that relies on the empirical null distribution, both for single-cell and bulk datasets. The empirical null strategy improved the FDR control in single-cell data and performed on par in bulk data, as shown in *Extended data* Figure S14. However, it is possible for the user to use the “raw” p-values and FDR instead, as they are also provided in the output of the *testDTU* function.
  The empirical null strategy being the default is discussed at the end of the “Performance on real single-cell data” paragraph of the Results section of the manuscript. However, in the Bioconductor vignette, we indeed failed to explicitly mention which strategy we are using. We have now added this information to the vignette, where we now also more explicitly describe the output of the *testDTU* function, i.e., the fact that both the raw and empirical p-values are returned and can both be used for downstream analysis (see the updated vignette at https://statomics.github.io/satuRn/articles/Vignette.html).
3. In the third comment, the reviewer requests to assess the effect of filtering and data sparsity on the performance of the different DTU tools. This relates to a comment also raised by reviewer #1, who requested to assess how the performance of the different DTU methods is affected by the percentage of zero counts in the data and if we could display some of the sparsity related summary statistics and distributions of the different benchmark datasets. We therefore address these comments jointly.
  We first provide a table ( *Extended data* Table S1) containing relevant summary statistics for the different datasets, including the number of features retained after filtering in the different datasets, as well as a figure (Figure S32) that visualizes some of the sparsity related properties of the datasets (e.g., library size, fraction zeros per cell). These clearly show that the Darmanis *et al.* dataset, for which the performance was lowest for each DTU method, is by far the sparsest dataset in our benchmark analyses.
  Second, we explored how differences in sparsity between the different datasets are reflected in the performance benchmarks. To this end, we construct FDR-TPR curves where the genes (not individual transcripts) have been stratified on the percentage of zero counts. For each dataset, we thus assess the performance on three strata of genes, i.e., for genes that have a low (< 25%), middle (25-50%) or high (> 50%) percentage of zero counts in their corresponding transcript-level count matrices. All the transcripts of the gene get stratified in this gene-level stratum. The results of this performance benchmark have been included in the *Extended data*, Figures S30 and S31. As expected, we observe that performances are higher when there is a low percentage of zero counts, uniformly across all datasets and for all methods. Second, performances on genes with high percentage zero counts increase strongly when the number of cells in the data increases (comparison between the bottom rows of Figures S30 and S31). Third, some methods seem more sensitive than others against sparse data. In particular, the performance of DEXSeq on the Tasic *et al.* 20v20 dataset and the Chen *et al.* 20v20 dataset (Figure S30) is on par with the performance of satuRn and DoubleExpSeq for the strata with a low or middle percentage of zeros, but markedly lower for the stratum with the highest percentage of zeros. As such, the performance gain of satuRn and DoubleExpSeq over DEXSeq in these datasets can be largely attributed to a better performance on the sparsest part of the data. Note that this is in line with the performance of DEXSeq being on par with satuRn and DoubleExpSeq on bulk data.
  In addition to *Extended data* Figures S30-S32 and Table S1, we included a discussion of these results in the Discussion section of the manuscript (third paragraph).
4. We have followed the reviewer’s suggestion of including an additional case study in the manuscript that performs a differential usage analysis on transcript compatibility counts (TCCs), sometimes referred to as equivalence class (EC) counts.
  More specifically, we have performed an EC-level analysis on the dataset of Tasic *et al.*, which was also used for the transcript-level case study, allowing us to qualitatively compare the results of both analyses. This additional analysis led to several novel insights which we describe detail in paragraph “Case study on transcript compatibility counts” of the Results section and discuss in the fourth paragraph of the Discussion section of the manuscript. In addition, Figure S27 was added to the *Extended data*. We here attempt to briefly summarise the key outcomes of the analysis.
  First, we display the number of differentially used transcripts and equivalence classes in eight comparisons between cell types in Table 3. The extent to which transcript-level and EC-level results correspond differs between the different comparisons. For several comparisons, there is a clear discrepancy between the number of differentially used transcripts and equivalence classes (Table 3, columns 4 and 6) and in the number of unique genes in which evidence for differential usage was observed (Table 3, columns 5 and 7). The overlap between the genes found in the transcript-level and the TCC-level analysis ranges from 53% (54/102) in comparisons 6 and 8 to 100% in comparison 3.
  For comparison 6 (53% overlap), we investigate the genes identified by both analyses. For some genes that are identified in both analyses, there is a strong agreement between the transcript-level and EC-level results. One example is the gene Pde4d. As depicted in Figure 11, there is a one-to-one concordance in the usage profiles of the equivalence classes and the transcripts. In other words, for this gene, the EC-level analysis is sufficient to obtain the same qualitative results as the transcript-level analysis. This is highly interesting, as the TCCs require only a fraction of the time to compute compared to the transcript-level counts, and they have no quantification uncertainty. However, for most other genes in which DU was identified in both analyses, the comparison between transcript and EC results is more ambiguous, as ECs often do not allow to pinpoint the responsible transcript(s) driving their observed DU. We demonstrate this by investigating the gene P2rx4. Four P2rx4 equivalence classes passed feature-level filtering, which we will denote with EC1-EC4. EC1 uniquely corresponds to transcript ENSMUST00000195963, and both the transcript and TCC are found to be differentially used in their respective analyses ( *Extended data* Figure S27, panel A). The other ECs correspond to two, three and four different transcripts, respectively ( *Extended data* Figure S27, panel A). For EC2 and EC3, strong evidence for differential usage was found. For EC4, there was no evidence for differential usage. However, based on these ECs alone, it would not be possible to unambiguously attribute these changes to single transcripts, complicating the biological interpretation.
  In the Discussion, we therefore conclude that biological interpretation of equivalence classes that correspond to multiple transcripts of a gene remains challenging. Currently, functional interpretation will therefore be limited to pinpointing genes with differential usage of a certain equivalence classes, without being able to link this to a functional effect at the transcript- or protein level.
  Finally, we note that while an analysis on scRNA-seq data from droplet protocols (like Chromium 10X) where the transcripts are not distinguishable from the 3’ ends alone would also be very interesting, we did not include such analysis in this manuscript. In our opinion, an extensive benchmarking of the different DTU tools on such data would be required and is out of the scope of this manuscript.
5. The reviewer asked if we by default use the stage-wise testing procedure stageR in a satuRn analysis workflow.
  We do not apply stageR by default. Hence, all results from the paper are generated without stage-wise testing to allow for a fair comparison between the methods. We did not systematically compare results for all methods with and without stageR, as this would further increase (double) the number of benchmarks in this paper. In addition, it is not an integral part of any DTU method and the utility of stageR in a DTU setting has been demonstrated previously in the original stageR publication of Van den Berge *et al*. However, conceptually, a stage-wise testing procedure makes sense in the context of DTU and should result in increased statistical power for all DTU methods. In this context, stageR has also been included in the downstream analysis of DTU methods in the F1000research workflow paper by Love *et al*. ( https://doi.org/10.12688/f1000research.15398.3).
  We only included stageR as a downstream analysis method in our package vignette, where we wanted to showcase how to use stageR in the downstream analysis of satuRn output. We have now specifically stated in the vignette that this is an optional step.
6. We have followed the reviewer’s suggestion of including an additional case study in the manuscript that performs a differential usage analysis at the level of exons. To briefly showcase this, we adopted the differential exon usage (DEU) analysis using the data from the DEXSeq vignette, i.e., we perform the same DEU analysis as the DEXSeq vignette using both DEXSeq and satuRn. This analysis has been included in the “Case study on exon-level counts” paragraph in the Results section of the manuscript. In addition, we added Figures S28 and S29 to the *Extended data.*
  Compared to the original DEXSeq analysis, satuRn identified fewer differentially used exons between the two experimental groups, with three statistically significant findings at 5% FDR for satuRn compared to 19 significant findings with DEXSeq. While we did not benchmark satuRn and DEXSeq on exon-level data, this could correspond to DEXSeq being overly liberal in transcript-level bulk analyses, especially with small sample sizes (Figure 2 and *Extended data* Figure S2). More importantly, however, the ranking of the exons based on their respective p-values is almost identical between the two methods ( *Extended data* Figure S28). Again, this is supportive of a similar performance of DEXSeq and satuRn on small bulk RNA-Seq datasets, albeit with a different type 1 error rate. In *Extended data* Figure S29, we additionally demonstrate how satuRn can be used to visualise changes in the usage of exons.
7. Finally, the reviewer request to improve the layout of the performance curves to reduce overplotting. Given that the performances of most methods are very close, this was not trivial. First, we did not want to reduce the range of the x-axis, as this would remove some of the FDR working points for the more liberal tools. Second, we prefer displaying the entire curve over only displaying the curve between the FDR working points of 0.01 and 0.1, which is also often done in this context. However, the latter strategy does not allow for displaying early mistakes, i.e., false positives with a very low FDR. Therefore, we aimed to accommodate the comment by reducing the size of the FDR working points, making the curves slightly thinner and by increasing the size of some of the plots.

# Peer review: Reviewer response for version 1

**DOI:** 10.5256/f1000research.54945.r85874

**Published:** 2021-06-10

**Authors:** Marek Cmero

**Affiliations:** 1 Walter and Eliza Hall Institute of Medical Research, Melbourne, Vic, 3052, Australia; 2 Department of Medical Biology, The University of Melbourne, Melbourne, Vic, 3052, Australia

Gilis *et al.* present satuRn, a novel method for performing differential transcript usage analysis on bulk and single-cell RNA-seq data. The approach improves upon existing methods through computational scalability and support for multifactorial experimental designs, while also tightly controlling FDR rates for bulk and single-cell experiments. The authors demonstrate performance using simulated, as well as real bulk and single-cell RNA-seq data, showing that satuRn performs on par with the best available approaches, while controlling the FDR and scaling favourably to large numbers of cells and transcripts. 

 The paper is well written, logical and easy to follow. The experimental methodology and results are sound and well presented. The authors have also done a great job with the software. I was able to install satuRn and run the vignette without any difficulty. 

 The following points would help improve the manuscript:

- The manuscript cites support for multifactor experimental designs as a key advantage of satuRn, however, this is not demonstrated. A small case study experiment with a multifactor experimental design, perhaps using the Tasic *et al*. data, would be helpful in illustrating this feature. 
- The authors benchmark satuRn's scalability on scRNA data, however, not on bulk RNA-seq (although competing methods are benchmarked on bulk data in Figure 1). While this is computationally less challenging, it would be helpful for the authors to also show satuRn’s scalability on bulk RNA-seq, for instance, using the Love *et al.* data as in Figure 1.
- The Discussion notes that performance on different scRNA datasets is affected by the percentage of zero counts: It would be helpful if the authors could show the zero count distributions in the three different scRNA data sets to illustrate this.
- It can be quite difficult to compare across the FDR-TPR curves when assessing performance using the different filters and abundance estimation methods. Perhaps for a subset of the comparisons and only showing the best performing tools, the authors could consider adding a set of simplified figures that compares the different filtering/counting approaches on a single plot per tool. 
- The authors state that performance of raw abundance estimates was better in all data sets, except for the Love *et al.* data., which is presumably due to the simulation methodology. Do the authors have any insights into why this dataset performed better using pseudo-alignments?
- The quantification methodology of the scRNA data is not described in the methods and should be included.
- In the two sections describing the real bulk and real single-cell study data, the authors refer to the ‘swapping strategy’ described in the Methods. However, two different swapping strategies are detailed. Please clarify.
- In the ‘Performance assessment’ section, the acronym FDP is not spelled out.
- Panels A-C in Figure 8 are missing labels.
- It would be helpful if the authors could explain the null distribution histograms a bit more, i.e. what the green and blue lines, red triangles and highlighted distributions show.

Is the rationale for developing the new method (or application) clearly explained?

Yes

Is the description of the method technically sound?

Yes

Are the conclusions about the method and its performance adequately supported by the findings presented in the article?

Yes

If any results are presented, are all the source data underlying the results available to ensure full reproducibility?

Yes

Are sufficient details provided to allow replication of the method development and its use by others?

Partly

Reviewer Expertise:

Bioinformatics, method development, transcriptomics

I confirm that I have read this submission and believe that I have an appropriate level of expertise to confirm that it is of an acceptable scientific standard, however I have significant reservations, as outlined above.

# Response

**Published:** 2022-07-25

**Authors:** Jeroen Gilis

**Affiliations:** Ghent University - VIB, Belgium

We are grateful for the comments and suggestions of both reviewers, which have markedly improved the readability of the manuscript and have led to several additional analyses and findings. We here first summarise the major and minor changes to the manuscript and then address the reviewer-specific comments point-by-point:

 Major changes:

- We include a case study analysis on single-cell RNA-seq data with a more complex multifactor experimental design. We first show that including additional covariates to the differential transcript usage (DTU) analysis affects the ranking of transcripts in the resulting top list as compared to an analysis without these covariates. Second, we use the more complex model to identify covariate-specific changes in DTU.
- We include a case study that performs a differential usage analysis at the level of equivalence classes. We qualitatively compare results between a transcript-level and equivalence class-level analysis on the same data, highlighting the advantages and disadvantages of both analyses.
- We included a short case study on differential exon usage on a small bulk RNA-seq dataset, comparing satuRn with DEXSeq.
- We elaborate on the effect of data sparsity and filtering on the benchmark performances of the different DTU tools. We additionally summarize the key characteristics of the different datasets.
- We elaborate on the comparison between using raw counts or scaledTPM abundances in the context of DTU analyses.
- We assess the performance of DTU methods as a function of the strength of the induced DTU signal as introduced by the swapping strategy in our performance benchmarks.
- We additionally include a scalability analysis of the different DTU tools on bulk RNA-seq data.

Minor changes:

- We discuss the discrepancy of the performance benchmark results between the dataset from Love *et al.* and the other datasets.
- We add information on the quantification strategies for the single-cell RNA-seq datasets.
- We make more explicit which strategies were used for introducing DTU signal in the different datasets that were used for benchmarking the DTU methods.
- We clarify the acronym FDP.
- We labeled the panels of Figure 8. In addition, we provided labels to Figure 6 and adapted the captions accordingly.
- We provide a more detailed description of the null distribution histograms in the *Extended data* figures.
- We clarify that, by default, satuRn adopts the testing strategy that is based on the empirical null distribution, but that users could opt for working with the unadjusted p-values as well.
- We clarify that we did not perform stage-wise testing adjustment in any of the analyses presented in the manuscript.
- We aimed to improve the layout of the performance curves.

Response to comments Reviewer 1:

1. We have followed the reviewer’s suggestion of including a case study analysis with a multifactor experimental design. We reanalysed the single-cell study from Tasic *et al* *.* but we now include a main effect for cell type, gender, and the continuous covariate age, as well as an interaction effect between cell type and gender. In this analysis, we first show that including these additional covariates affects the ranking of transcripts in the resulting top list as compared to our original unadjusted analysis. Second, we assessed the interaction effect to prioritise transcripts for which the DTU between cell types differs according to gender. Here, we highlight a transcript that displays strong evidence for DTU between cell types in female mice, but not in male mice. The analysis has been included in the manuscript as a new paragraph, “Analyses with complex experimental designs”, under the Results section and includes the new Figure 10.
2. We have followed the reviewer’s suggestion of including an additional scalability benchmark on bulk data. We performed the benchmark on the GTEx dataset and not the Love dataset (as was suggested by the Reviewer), because the latter only contains 24 samples. The results of the benchmark are in line with those of the single-cell scalability benchmark, with DEXSeq and DRIMSeq being markedly slower than the other methods. When directly comparing the scalability between the bulk and single-cell data on equally sized subsets, we found limited differences in scalability between the data types, except for DEXSeq. DEXSeq scales considerably better on bulk data than on single-cell data, suggesting that the estimation of the GLM parameters is slower when the data are sparse. However, since the scalability profile of DEXSeq is quadratic with respect to the number of cells/samples in the data, it is still infeasible to adopt DEXSeq in bulk datasets with many samples. The discussion of the results has been added to the “Scalability benchmark” paragraph of the Results section. Figures of the scalability profiles on bulk data have been included in the *Extended data*, Figures S20 and S21.
3. In the third comment, the question was raised as to how the performance of the different DTU methods is affected by the percentage of zero counts in the data. This relates to a comment raised by reviewer #2, who requested “to show [DTU] benchmarks with less stringent criteria to assess how the presence of many zeros affect [the performance of] each method”. We therefore address these comments jointly.
  We provide *Extended data* Table S1 containing relevant summary statistics for the different datasets, including the number of features retained after filtering, as well as Figure S32 that visualizes some of the sparsity related properties of the datasets (e.g., library size, fraction zeros per cell). These clearly show that the Darmanis *et al.* dataset, for which the performance was lowest for each DTU method, is by far the sparsest dataset in our benchmark analyses.
  Second, we explored how differences in sparsity between the different datasets are reflected in the performance benchmarks. To this end, we construct FDR-TPR curves where the genes (not individual transcripts) have been stratified based on the percentage of zero counts. For each dataset, we thus assess the performance on three strata of genes, i.e., for genes that have a low (< 25%), middle (25-50%) or high (> 50%) percentage of zero counts in their corresponding transcript-level count matrices. All the transcripts of the gene get stratified in this gene-level stratum. The results of this performance benchmark have been included in the *Extended data*, Figures S30 and S31. As expected, we observe that performances are higher when there is a low percentage of zero counts, uniformly across all datasets and for all methods. Second, performances on genes with high percentage zero counts increase strongly when the number of cells increases (comparison between the bottom rows of Figures S30 and S31). Third, some methods seem more sensitive than others against sparse data. In particular, the performance of DEXSeq on the Tasic *et al.* 20v20 dataset and the Chen *et al.* 20v20 dataset (Figure S30) is on par with the performance of satuRn and DoubleExpSeq for the strata with a low or middle percentage of zeros, but markedly lower for the stratum with the highest percentage of zeros. As such, the performance gain of satuRn and DoubleExpSeq over DEXSeq in these datasets can be largely attributed to a better performance on transcripts of lowly abundant genes. Note that this is in line with the performance of DEXSeq being on par with satuRn and DoubleExpSeq on bulk data.
  In addition to *Extended data* Figures S30-S32 and Table S1, we included a discussion of these results in the Discussion section of the manuscript (third paragraph).
4. We have followed the reviewer’s suggestion of including simplified figures that compare the different filtering and counting approaches on a single plot per tool. These figures have been included as *Extended data* Figures S10-S13 and are discussed in the “Performance on simulated bulk RNA-seq datasets” paragraph in the Results section of the manuscript.
  These figures show more clearly that neither sample size nor the filtering strategy had a profound impact on the ranking of the performances of the different DTU methods; satuRn, DEXSeq and DoubleExpSeq remain the best performing methods overall. However, we did find that the effects of filtering and counting differ between the different datasets.
  For the Love *et al.* simulated bulk dataset, filtering more stringently strongly improves performance. In addition, both performance and FDR control are much better when using scaledTPM abundances, as compared to using counts. The strong positive effect of using scaledTPM abundances are only observed for this dataset, which we will discuss in more detail in our response to the next comment of the reviewer.
  For the simulated bulk datasets by Van den Berge *et al.*, we also observe a positive effect of stringent filtering. The difference between scaledTPM and raw count abundances is negligible.
  For the real datasets, a quite different pattern is observed. For the GTEx bulk dataset and the single-cell datasets by Tasic *et al.* and Chen *et al.*, the effects of filtering are limited and using counts performs slightly better than using scaledTPM abundances.
  Finally, stringent filtering had a strong positive impact on performance on the Darmanis *et al.* dataset. Note that this dataset is by far the sparsest dataset in our benchmarks ( *Extended data* Figure S32 and Table S1). The differences in performance between scaledTPM and raw count abundances are small.
  Taken together, using scaledTPM abundances is only beneficial in the simulations by Love *et al.*, for reasons we explain in below. Therefore, we recommend using raw counts abundances. With respect to filtering, the more stringent DRIMSeq filtering strategy only seems beneficial in simulated data and in very sparse single-cell data. Thus, for bulk data and regular single-cell data the lenient edgeR filtering strategy seems sufficient to increase the signal-to-noise ratio.
5. In our hands, the use of scaledTPM abundances instead of raw counts was indeed only beneficial in the simulated dataset by Love *et al.* As suggested by the reviewer, this is indeed due to the simulation methodology.
  Love *et al.* introduced DTU either by swapping TPM abundances or by introducing a fold change in transcript expression in one group of samples, again on TPM abundances. However, when back-transforming the swapped TPM abundances to count abundances, the transcript lengths were not swapped. Hence, the simulation strategy can induce differences in the total count for a gene, which in turn may alter the usage of all (target and off-target) transcripts in that gene when raw counts are used as an input, which gives rise to many false positives in off-target transcripts of DTU genes.
  When introducing DTU by swapping raw counts (real bulk and single-cell evaluations), we could expect spurious signal in analyses on the TPM scale for the same reason. However, the difference between the raw count and TPM analyses was much less pronounced, which was also the case for the Van den Berge *et al.* simulation approach.
  We have included this as a remark in the “Performance on simulated bulk RNA-seq datasets” paragraph in the Results section of the manuscript.
6. The quantification methodology for the single-cell RNA-seq data was indeed missing and has now been included in the “Real single-cell study” paragraph of the Methods section as follows: “The datasets from Chen *et al*. ^29^ and Tasic *et al*. ^42^ were quantified using Salmon v1.1.0, whereas the dataset from Darmanis *et al*. ^26^ was quantified with Salmon v0.8.2 as obtained from Soneson et al. ^43^.”
7. We agree with the reviewer that it previously unclear which swapping strategy was used in the “real bulk study” and “real single-cell study” paragraphs of the Methods section. In both cases, DTU is artificially introduced by swapping raw transcript counts between groups of samples, which we now explicitly mention in both paragraphs.
8. We have now spelled out the acronym FDP in the “Performance assessment” paragraph of the Methods section, and defined it as follows: “The FDR is the expected value of the false discovery proportion (FDP), which is the ratio of the number of false positives over the total number of positives.”
9. As requested, we labeled the panels of Figure 8. In addition, we provided labels to Figure 6 and adapted the captions accordingly.
10. We agree with the reviewer that the null distribution histograms in the Extended data Figures S14, S15, S24 and S26 required further explanation. We have included this information in the caption of Figure S14 and referred to that information in the captions of Figures S15, S24 and S26. The update caption of Figure S14 reads as follows: “The z-scores corresponding to the null transcripts are expected to follow a standard normal distribution (mean = 0, standard deviation = 1). This corresponds well with the maximum likelihood estimates (MLE) for the mean and variance of the empirical null distribution (mean = -0.002, standard deviation = 1.029) as obtained with the locfdr package. In brief, these estimates are obtained by assuming that the z-scores of all transcripts follow a mixture distribution, where the z-scores of the null transcripts are expected to follow a normal distribution and the z-scores of the DTU transcripts follow some other distribution. Two models are fitted to the z-scores. The blue dashed curve is a normal distribution that is fitted to the mid 50% of the z-scores, which are assumed to originate from null genes, thus representing the estimated empirical null component densities. The MLE and central matching estimates (CME) for the mean and standard deviation of the estimated empirical null distribution are provided in the caption at the bottom of the plot. Finally, the green solid curve represents the estimated marginal density across all z-scores and is obtained by fitting a spline model to the histogram counts.”
  To avoid confusing the reader with concepts that are not required for the paper, we have omitted the explanation of the yellow triangles on the *locfdr* figures. In brief, the *locfdr *package returns local fdr values for each transcript. The FDR is a set property, i.e., the expected fraction of false positives in the set that is returned. Efron (2010) showed that the FDR can also be interpreted as a posterior probability, i.e., the posterior probability that a random feature in the set that is returned is a false positive. Unlike the FDR, the local fdr of a feature is the posterior probability that this individual feature is a false positive, so it relates to a single specific null hypothesis of this specific feature and not to a set of null hypotheses. Efron (Ann. Stat. 2007) defined 0.2 as a useful cutoff for the local fdr and argues that a 0.2 cutoff for the local fdr often corresponds with a conventional FDR of 0.05. Efron showed that the FDR can also be interpreted as the average local fdr over the set that is returned as significant, i.e., the features with a local fdr < 0.2. The conventional FDR over this set can be estimated by taking the sample average of the local fdr of the features that are returned, and is bound to be much smaller than 0.2.  The triangles on the *locfdr* plot indicate cut points for the *z*-scores; *z*-scores that are more extreme than the triangles will result in a local FDR estimate that is smaller than 0.2.
